# Differential Changed Excitability of Spinal Motor Neurons Innervating Tibialis Anterior and Peroneus Muscles Cause Foot Inversion After Stroke

**DOI:** 10.3389/fneur.2020.544912

**Published:** 2020-11-24

**Authors:** Gang Liu, Chin-hsuan Chia, Yue Cao, Xin-wei Tang, Shan Tian, Xue-yan Shen, Ying Chen, Rong-rong Lu, Jun-fa Wu, Yi Wu

**Affiliations:** Department of Rehabilitation Medicine, Huashan Hospital, Fudan University, Shanghai, China

**Keywords:** stroke, spinal motor neuron, excitability, muscle, motor recovery

## Abstract

**Objective:** To study differential post-stroke changes of excitability of spinal motor neurons innervating a group of antagonist muscles of ankle and their effects on foot inversion.

**Methods:** F waves in tibialis anterior (TA) and peroneus muscles (PN) were recorded. The condition of spasticity and foot inversion in stroke patients were also evaluated. The differences of F wave parameters between patients and healthy controls (HC), as well as TA and PN, were investigated.

**Results:** There were natural differences in the persistence of the F waves (Fp) and F/M amplitude ratio (F/M) between TA and PN in HC. Stroke patients showed significantly higher F/M in TA and PN, while there was no difference in Fp comparing to HC. The natural differences in F wave parameters between TA and PN were differentially retained after stroke. The natural difference of the two muscles in Fp remained unchanged and the F/M difference disappeared in those without spasticity or foot inversion, while the Fp difference disappeared and the F/M difference remained in those with spasticity or foot inversion.

**Conclusion:** Based on the natural difference of the number and size of spinal motor neurons innervating TA and PN, their excitability may change differently according to the severity of the stroke, which may be the reason of foot inversion.

## Clinical Messages

1. Natural differences in F wave parameters between tibialis anterior and peroneus muscles exists.
2. The F/M ratio of both muscles increased, while F persistence remained unchanged after stroke.
3. Based on the natural difference of the number and size of spinal motor neurons innervating TA and PN, their excitability may change differently according to the severity of the stroke, which may be the reason of foot inversion.

## Introduction

Motor dysfunction after stroke is the main cause of disability, and still a major challenge to neurological rehabilitation (1). In clinical practice, there is an obvious disparity in velocity and extent of recovery among different paralyzed muscles after stroke (2, 3). Some muscles, such as the elbow flexor, are easily able to recover even in severe stroke; while other muscles, such as the peroneus, often show persistent weakness. The reason for this phenomenon remains elusive, deserving further researches on its pathophysiological mechanism. It is well known that the recovery of motor function is closely related to the spinal neural excitability (4, 5). After stroke occurs, motor neurons with relatively higher excitability tend to be excited by the residual descending impulses of the pyramidal tracts or extrapyramidal systems, causing voluntary contraction or spasticity of the corresponding muscles (6), while motor neurons with relatively lower excitability are unable to be excited and remained paralyzed. Therefore, we hypothesized that the excitability of spinal motoneurons dominating different muscles will change differently after stroke.

Most of the previous researches (7–9) have focused on the change of spinal motor excitability of one specific muscle but have rarely compared the difference of the excitability between a couple of antagonistic muscles after stroke and its potential influence on motor dysfunction. Given the high incidence of foot inversion in stroke patients (10), we intentionally selected TA and PN as target muscles, which are biomechanically antagonistic to each other (i.e., TA for ankle dorsiflexion and foot inversion, while PN for plantar flexion and foot eversion). By analyzing the excitability changes of the two muscles, we intend to discuss the difference of changed excitability between TA and PN, and moreover, the influence on foot inversion in stroke patients.

F wave is a late electrophysiological response generated by a supramaximal electrical stimulus that triggers impulse retrograde along the axon activating the motor neuron in anterior horn and then returns anterograde along the same axon to the corresponding muscle. It is widely considered as a reflection of the spinal motor neuron excitability (11, 12). In this study, we intended to stimulate the common peroneal nerve which innervating both TA and PN and recorded the F wave of the two muscles. By comparing the difference of the F wave parameters between TA and PN, the deviation of the excitability of the spinal motoneurons can be reflected.

## Methods

### Trial Design

This study was designed as a prospective cohort observational study, strictly followed the STROBE guideline (13). The clinical scales and F wave measurement were conducted on the subjects. This study was approved by the Huashan hospital Ethics Committee and was registered on the Chinese Clinical Trial Registry (ChiCTR1800016212).

### Participants

According to the result of sample size calculation, thirty-three stroke patients (27 males and six females, age: 52.45 ± 1.95) were recruited from Huashan Hospital affiliated to Fudan University, from July 2019 to October 2019 (see Table 1). Stroke was defined as an acute episode of focal dysfunction of the brain, retina, or spinal cord lasting longer than 24 h, or any duration if imaging or autopsy showed focal infarction or hemorrhage relevant to the symptoms (14). Patients were strictly chosen according to the diagnostic criteria of the stroke to eliminate the selection bias. Stroke patients were included if they met the following criteria. (1) 30–75 years old; (2) first-ever cerebrovascular episode accompanied with extremity motor disorder; (3) duration more than 1 month; (4) absence of complications (consciousness, speech, cognitive, or psychotic disorders, etc.) which might influence the proceeding of our investigation; (5) absence of peripheral neuropathy or spinal cord injury; (6) absence of passive ankle movement disorder.

**Table 1:** Basic information of the patients.

	**Stroke patients**	**Healthy controls**	***p***
Number	33	25	
Age, years	52.45 ± 1.958	49.80 ± 9.967	>0.05
Gender (male/female)	27/6	17/8	>0.05
Duration, days	162.1 ± 36.68		
CSS evaluation (no-mild/medium-severe)	9/24		
Foot inversion (without/with)	12/21		
dorsiflexion without/with foot inversion	5/19		

Twenty-five healthy participants (17 males and eight females, age: 49.80 ± 9.967) were recruited for the control group. There was no statistical difference in the age and gender ratio between stroke patients and the control group (*p* > 0.05) (Table 1). Written informed consent for participation was obtained from all participants.

### Clinical and Demographic Measures

Age, gender, diagnosis of disease, hemiplegia side and the course of the disease were recorded in the stroke patients. The composite spasticity scale (CSS) (15), which assesses the severity of the spasticity of the lower extremity based on achilles tendon reflex, muscle tone and ankle clonus, was calculated to demonstrate the status of spasticity in the lower extremity. We documented whether there is active dorsiflexion of the ankle which was defined as the patient's ability to lift the planta pedis off the ground voluntarily. We also recorded the presence or absence of foot inversion in stroke patients. Subjects who exist foot inversion at either resting state or active dorsiflexion state were classified as foot inversion in our study. To distinguish the existence of foot inversion, videos were taken in both resting and active states. The identification was proceeded by two evaluators without knowing each other's judgments and only when both their opinions on foot inversion were identical, the existence of foot inversion was determined (10). When the evaluation, the subjects sat relaxed on the chair, knees flexed to 90 degrees and placed the planta pedis on the ground. The same researcher completed all evaluations for each patient.

### Recording of F Wave

**Figure 1:**
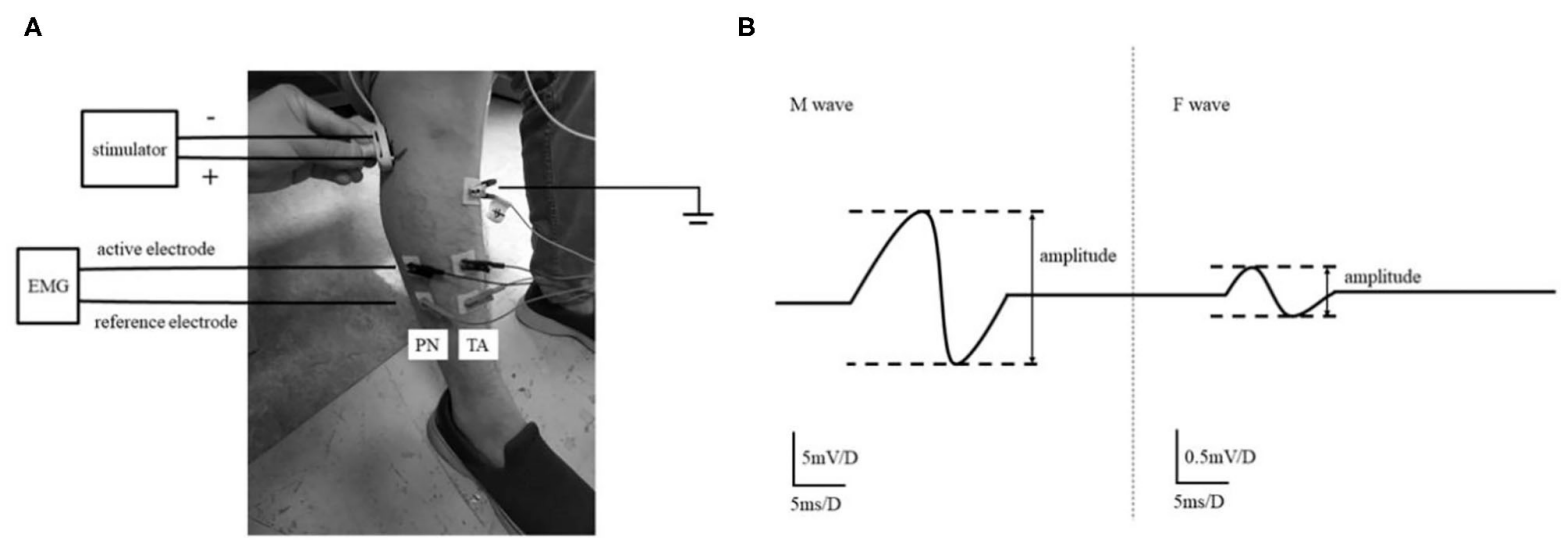
The experimental setup. The setting of F wave recording of TA and PN **(A)**. The study recorded the F wave of TA and PN that both of them are innervated by Common Peroneal nerve. M-wave and F-wave analysis **(B)**. Amplitude ratio of F/M was the ratio of the average F-wave amplitude and the average M-wave amplitude in the signal plot.

Before measuring F waves, participants were required to relax at least for 5 min. The subjects sat on the chair comfortably, with the knee flexed to 90° and planta pedis placed on the ground. Surface electromyography (EMG) recording was obtained from TA and PN. EMG signals were obtained through adhesive Ag-AgCl electrodes. The bipolar EMG electrodes were attached over the TA and PN. The active electrode of TA was on the belly of the muscle (10 cm below the tubercle of tibia and 1 cm lateral tibia), and the PN's active electrode was placed 10 cm below the fibulae capitulum. The reference electrodes were 2 cm distal from each active electrode. The ground electrode was placed 0.5 cm below the tubercle of tibia. The electrical stimulations transferred to the common peroneal nerve 0.5 cm posterior the fibulae capitulum via bipolar surface stimulator with the cathode being placed proximally (Figure 1A). The supramaximal stimulation (40 mA, 0.2 ms) was repeated 20 times at a frequency of 0.5 Hz. All signals were processed using NeuroCare-C (Shanghai NCC Electronic Co., Ltd., China) with a band-pass filter of 20 Hz−3 kHz, and the sensitivity was set and 0.5 mV per division for recording M waves and F waves (16), respectively (Figure 1B). The F waves were obtained from both lower extremities in every participant. The measurements were performed on the same day when the clinical measurements were done.

The persistence of the F waves (Fp) and F/M amplitude ratio (F/M) were obtained. Valid F waves were recognized as the potential with a peak-to-peak amplitude of at least 0.1 mV. Fp is the number of definable F responses per 20 stimuli, expressed as a percentage. Peak to peak amplitudes of F- and M-waves were measured, and the amplitude ratio of F/M was expressed as the ratio of F amplitude and the amplitude of M-wave.

### Data Analysis

1. We compared the same muscle's F wave among groups [e.g.,: compare the TA's F wave among healthy controls and affected limbs(A) of stroke patients] in order to reflect the change in the excitability of spinal cord neurons which control the same muscle after stroke.

2. In this study, the results of the F waves were classified and analyzed to compare the excitability of spinal motor neurons based on the different levels of spasticity and the existence of the foot inversion. The spasticity of the stroke patients was classified into two groups according to CSS score, the no/mild spasticity group (CSS below 10) and moderate-severe spasticity group (CSS above 10).

3. The change rate of spinal motor excitability after stroke. We calculated the change rate as:

$$\begin{array}{l} changerate=\frac{\left(\bar{patients^{\prime }}-\bar{controls^{\prime }}\right)}{\bar{controls^{\prime }}} \end{array}$$

The change rate of TA and PN was also compared in affected limbs of stroke patients.

4. In order to eliminate the effect of gravity on foot inversion caused by the biomechanics characteristics of the ankle joint when both the TA and PN were paralyzed (foot drop with foot inversion), we deliberately analyzed the ability of voluntary ankle dorsiflexion stroke patients.

### Statistically Analysis

All statistical analysis was performed with GraphPad Prism version 6.01. As for continuous variables of the baseline, results were presented as mean ± standard error of the mean (SEM) in the text and figures.

The Chi-square test was used to analyze comparisons of categorical variables. Comparisons of continuous variables were analyzed by one-way analysis of variance (ANOVA) or nonparametric test (Wilcoxon test or Mann Whitney test according to whether the two group's was paired or not) according to the number of groups (2 groups: nonparametric test, >2group: one-way ANOVA). One-way ANOVA was carried out to proceed stratified analysis about the same muscles, for example, TA's F/M in healthy controls, patients with inversion, and patients without inversion. When the proof in influence F-parameters was significant (*p* < 0.05), *post hoc* multiple comparisons using the Tukey test were performed.

## Results

**Figure 2:**
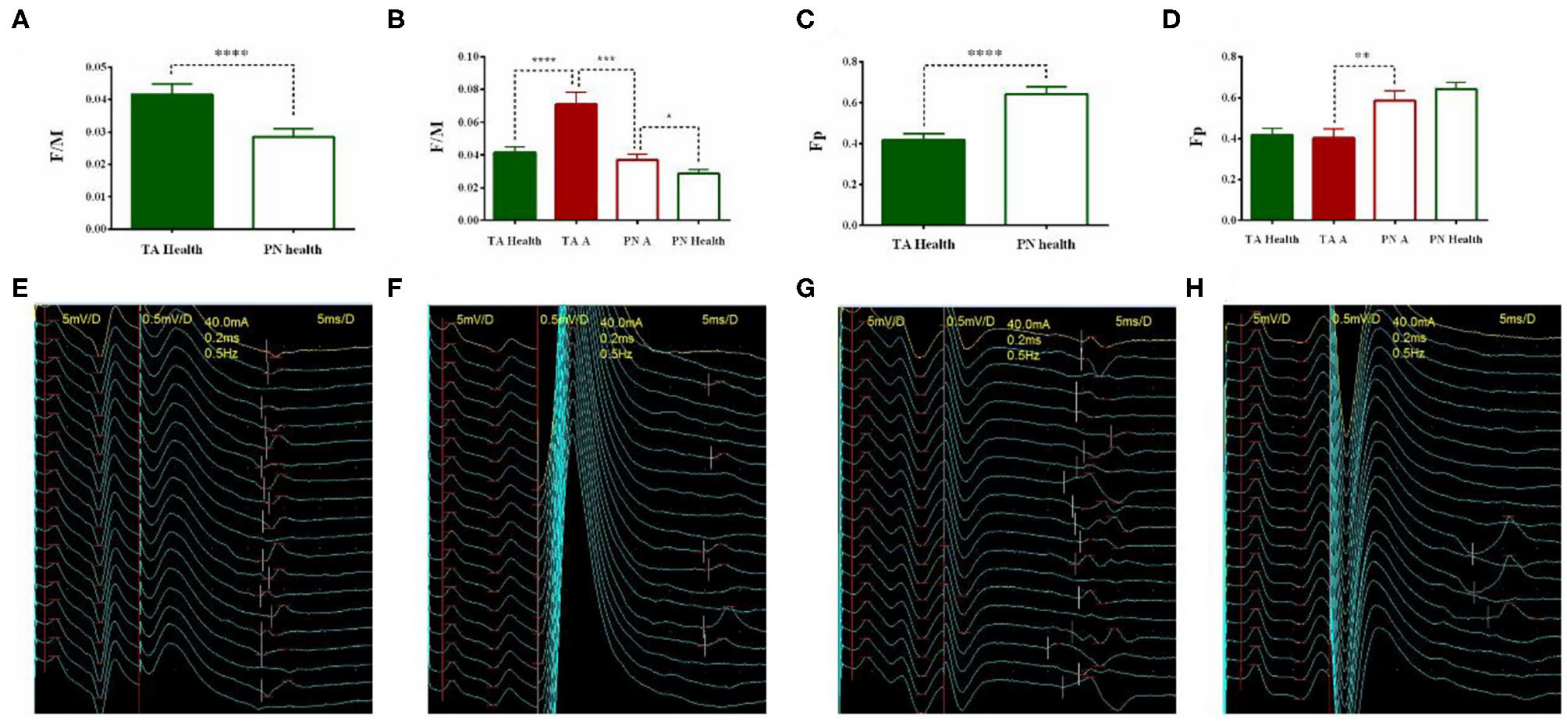
The natural difference of F wave between TA and PN and the change after stroke. The natural differences of F/M between TA and PN in healthy controls **(A)**. The F/M of TA and PN of the affected side increased significantly after stroke **(B)**. The natural differences of Fp between TA and PN in healthy controls **(C)**. The Fp of both TA and PN remained unchanged after stroke **(D)**. Typical image of F wave examination of PN (**E**, high Fp, low F/M) and TA (**F**, low Fp, high F/M) in healthy group, and PN (**G**, F/M increased significantly while Fp unchanged) and TA (**H**, F/M increased significantly while Fp unchanged) in stroke patients (*p < 0.05, **p < 0.01, ***p < 0.001, ****p < 0.0001; error bar:1 SEM).

There was a fundamental difference between the TA and PN's F/M in the healthy group (TA: 0.0416 ± 0.0032; PN: 0.0287 ± 0.0025; Z = −4.233, *p* < 0.0001) (Figure 2A). The average of TA's F/M was 1.5 times higher than the PN's. Meanwhile, there also existed the fundamental difference in Fp between TA and PN (TA: 0.4173 ± 0.0325; PN: 0.6408 ± 0.0355; Z = −4.644, *p* < 0.0001) (Figure 2C). TA's Fp was approximately two-thirds of the PN's. So, there was no difference between the TA and PN's producing of the F/M and Fp (TA health: 0.0191 ± 0.0026; PN health: 0.0183 ± 0.0019; Z = −0.264, *p* = 0.7977).

Both the F/M of the TA and PN in the stroke patients were significantly higher than the healthy controls (TA patients: 0.0710 ± 0.0073, Z = −4.065, *p* < 0.0001; PN patients: 0.0369 ± 0.0037, Z = −2.135, *p* = 0.0474 < 0.05) (Figure 2B). There was no difference between the stroke patients and the healthy controls' Fp of the TA and PN (TA patients: 0.4030 ± 0.0456, Z = −0.422, *p* = 0.6425; PN patients: 0.5848 ± 0.0485, Z = −0.936, *p* = 0.3796) (Figure 2D).

The fundamental differences of the F/M and Fp in TA and PN were reserved after stroke. The TA's F/M was significantly higher than the PN's (Z = −3.507, *p* = 0.0002 < 0.001) and the fundamental difference tended to be intensified (Figure 2B). However, the TA' Fp was significantly lower than the PN's (Z = −2.699, *p* = 0.0063 < 0.01) and the fundamental difference tended to be reduced (Figure 2D). Typical images were showed in Figures 2E–H.

### The Stratified Analysis Based on CSS

**Figure 3:**
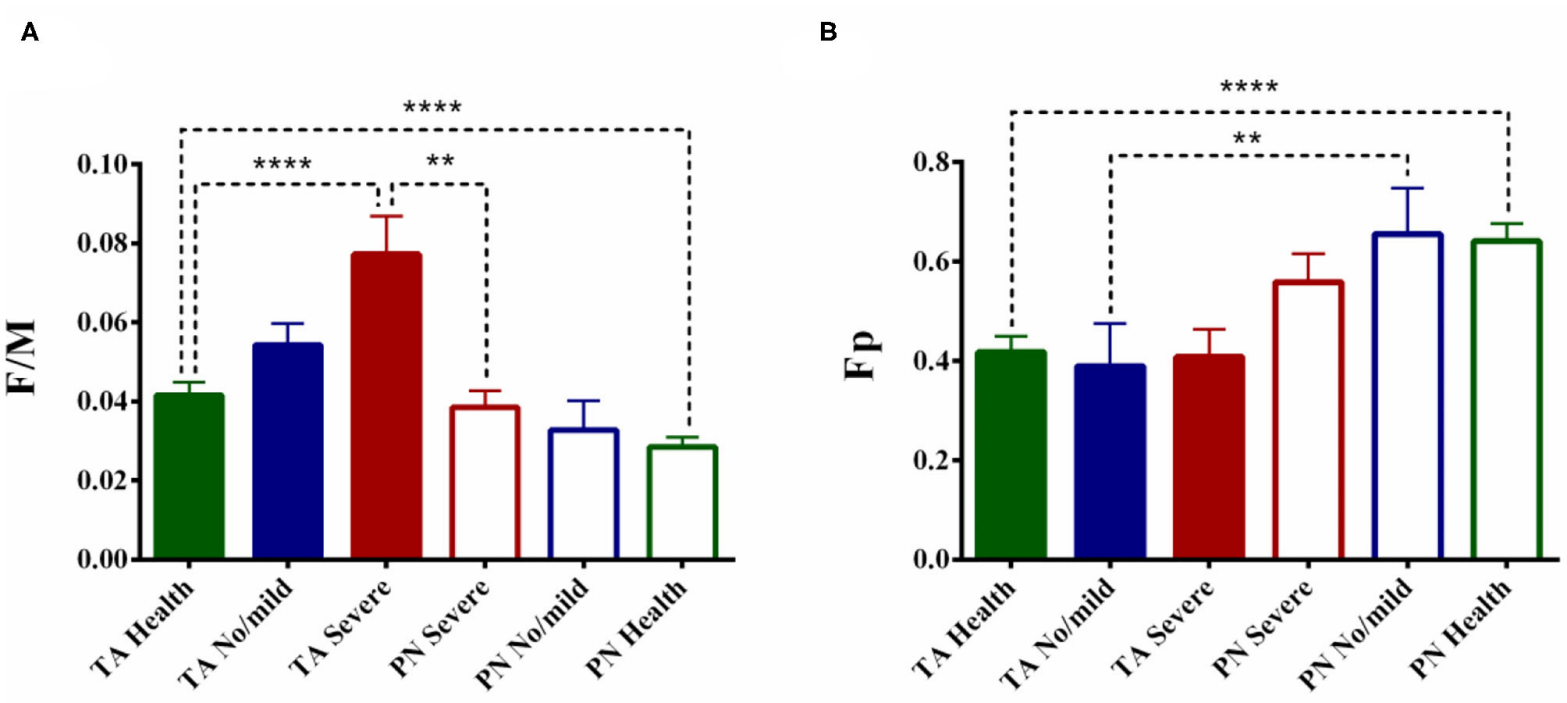
The stratified analysis based on spasticity. The comparison of F/M **(A)** and Fp **(B)** based on spasticity. The spasticity of the stroke patients was classified into two groups according to their results of CSS, the no/mild spasticity (CSS below 10) and moderate-severe spasticity (CSS above 10). (**p < 0.01, ****p < 0.0001; error bar:1 SEM).

The average data showed the F/M of TA differs significantly among the three groups (i.e.,: patients with medium/severe spasticity, patients with no/mild spasticity, and healthy controls)while the F/M of PN has no statistical difference among the groups (TA: F_2, 79_ = 9.119, *p* < 0.0001; PN: F_2, 79_ = 2.203, *p* = 0.1172). *Post hoc* comparisons revealed the F/M of TA was significantly higher in the patients with medium/severe spasticity than the healthy controls (TA patient severe: 0.0772 ± 0.0096, *p* < 0.0001). There was no statistical difference in the patients with no/mild spasticity (TA patient no/mild: 0.0543 ± 0.0054, *p* = 0.51) (Figure 3A). For Fp, the one-way ANOVA failed to show any statistical significance in both TA and PN (TA: F_2, 79_ = 0.2317, *p* = 0.7937; PN: F_2, 79_ = 0.4670, *p* = 0.6286) (Figure 3B).

In the group of no/mild spasticity, the fundamental difference between the TA and PN retained in the Fp but disappeared in F/M (Fp: Z = −2.524, *p* = 0.012 < 0.05; F/M: Z = −1.836, *p* = 0.066), while in patients with medium/severe spasticity, the fundamental difference of the F/M still existed but the differences in Fp disappeared (Fp: Z = −1.797, *p* = 0.072, F/M: Z = −3.027, *p* = 0.002 < 0.01).

### The Stratified Analysis Based on Foot Inversion

**Figure 4:**
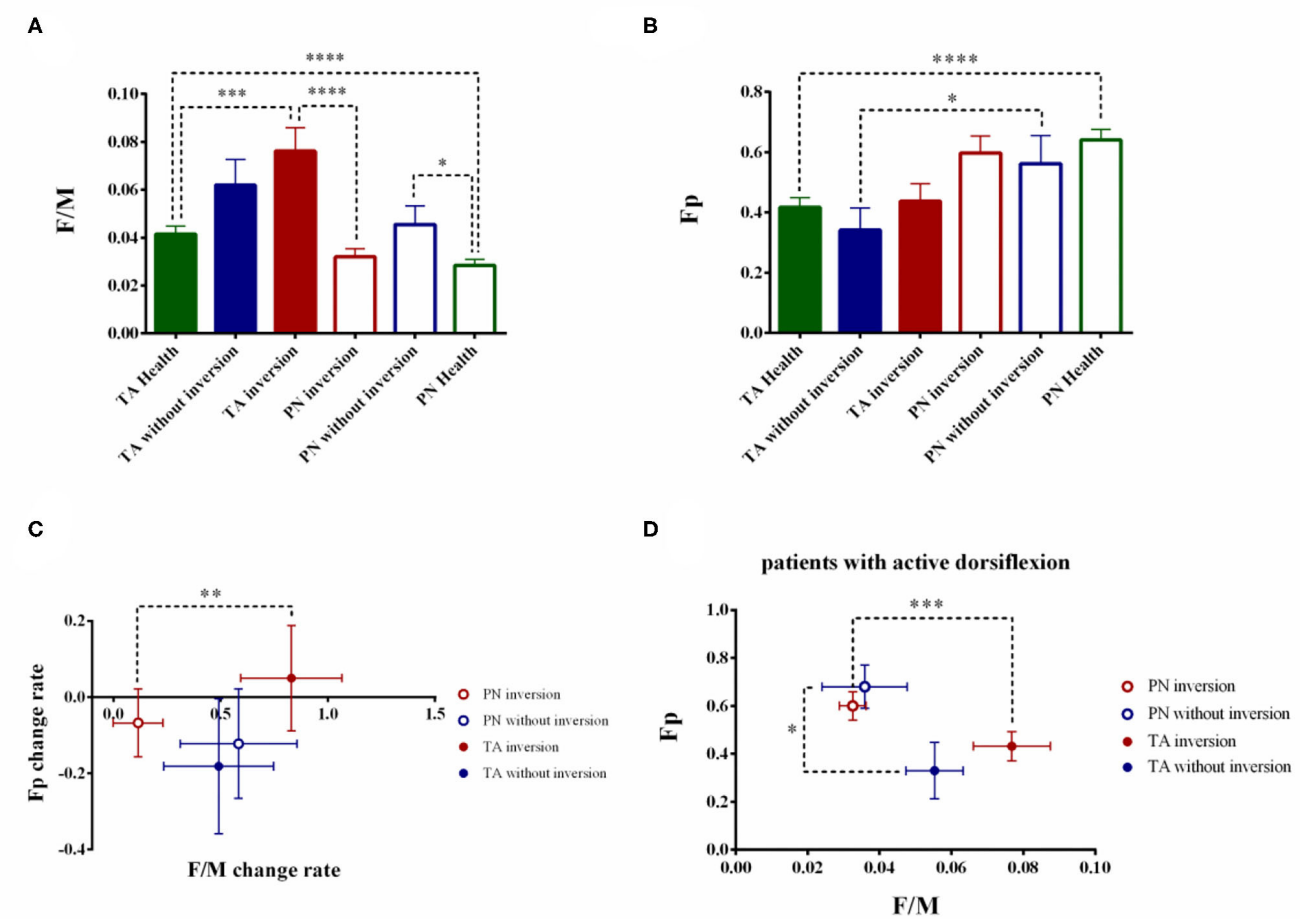
The stratified analysis based on foot inversion. The comparison of F/M **(A)** and Fp **(B)** based on foot inversion. The stroke patients were divided into two groups based on the foot inversion. The change rate of the F/M and Fp were also calculated **(C)**. To eliminate the interfered of the biomechanics factor, the patients with active dorsiflexion were extracted and the stratified analysis according to the foot inversion were conducted. **(D)** (*p < 0.05, **p < 0.01, ***p < 0.001, ****p < 0.0001; error bar:1 SEM).

F/M differed significantly among the three groups' TA and PN (TA: F_2, 79_ = 5.384, *p* = 0.0003; PN: F_2, 79_ = 3.990, *p* = 0.0223). *Post hoc* comparisons showed patient with inversion had higher F/M in TA than the healthy group (TA patient with foot inversion: 0.0761 ± 0.0098, *p* = 0.0002 < 0.001) while patient without inversion had higher F/M in PN than the healthy controls (PN without foot inversion: 0.0455 ± 0.0078, *p* = 0.0163 < 0.05) (Figure 4A). Statistical analysis showed no significant difference in Fp in both muscle among three groups (TA: F_2, 79_ = 0.2629, *p* = 0.7695; PN: F_2, 79_ = 1.130, *p* = 0.3283) (Figure 4B).

The fundamental difference of the TA and PN's F/M retained in the patients with foot inversion (PN patient with foot inversion: 0.0320 ± 0.0034; Z = −3.598, *p* < 0.0001) while disappeared in the patients without foot inversion (TA patients without foot inversion: 0.0620 ± 0.0107; PN patients without foot inversion: 0.0455 ± 0.0078; Z = −0.845, *p* = 0.398). On the contrary, the fundamental difference between the TA and PN's Fp retained in the patients without foot inversion (TA patients without foot inversion: 0.3417 ± 0.0741; PN patient without foot inversion: 0.5625 ± 0.0921; Z = −2.315, *p* = 0.021 < 0.05) but disappeared in the patients with foot inversion(TA patients with foot inversion: 0.4381 ± 0.0578; PN patient with foot inversion: 0.5976 ± 0.0921; Z = −1.910, *p* = 0.056).

In the patients without foot inversion, the change rate of the F/M and Fp showed no difference between TA and PN (F/M TA: 49.03 ± 25.59%; F/M PN: 58.32 ± 27.28%; Z = −0.157, *p* = 0.875; Fp TA: −18.12 ± 17.75%; Fp PN: −12.22 ± 14.38%; Z = −0.314, *p* = 0.754) (Figure 4C). In the patients with foot inversion, the F/M's change rate of the TA was significantly higher than the PN's (TA: 82.99 ± 23.59%; PN: 11.44 ± 11.66%; Z = −2.728, *p* = 0.006 < 0.01) while the change rate of Fp in TA and PN had no difference (TA: 4.98 ± 13.86%; PN: −6.74 ± 8.87%; Z = −0.330, *p* = 0.741).

### The Stratified Analysis Based on Foot Inversion in Patients With Voluntary Ankle Dorsiflexion

Totally 24 patients showed active ankle dorsiflexion and 19 of them were accompanied with foot inversion. In the patients with foot inversion and dorsiflexion, the TA's F/M showed significant increase than the PN's (TA: 0.0768 ± 0.0107; PN: 0.0325 ± 0.0037; Z = −3.381, *p* = 0.0002 < 0.001), while Fp in these two muscles showed no difference (TA: 0.4316 ± 0.0610; PN: 0.6000 ± 0.0594; Z = −1.902, *p* = 0.057) (Figure 4D). In the patients with ankle dorsiflexion but without foot inversion, the TA and PN's F/M showed no difference (TA: 0.0553 ± 0.0080; PN: 0.0359 ± 0.0118; Z = −0.944, *p* = 0.345 > 0.05), but the PN's Fp was significantly higher than the TA's (TA: 0.3300 ± 0.1179; PN: 0.6800 ± 0.0903; Z = −2.023, *p* = 0.043 < 0.05).

Whether patients with voluntary ankle dorsiflexion or not, the stratified analysis based on foot inversion presented the same result which indicated that the difference between the two muscles was attributed to spinal excitability difference rather than the biochemical characters of the ankle.

## Discussion

It is a common phenomenon that different muscles show deviation in the degree of motor recovery in stroke patients (17). The difference in recovery may reflect the excitability difference of spinal motor neurons after stroke. The neuro-electrophysiological examination is a convenient method to evaluate spinal motor excitability. It is believed that F waves are superior to H reflexes in evaluating spinal excitability because they are independent of sensory afferents and can be induced in most of muscles (11, 18–20). According to the mechanism how F wave generates, we considered that Fp reflects the number of excitable motor neurons that reach the set threshold of F wave (21), while F/M represents both the number of excitable motor neurons and the average volume of the motor unit (MU) they govern (20). Both of them are an indicator of the excitability of motor neurons, and that is the reason why the two parameters are selected for analysis in this study.

In healthy controls, we found that there was a natural difference between TA and PN that TA's F/M is higher and Fp is lower. This phenomenon may be caused by a higher proportion of large MUs with lower excitability in TA than in PN. It is generally believed that the recruitment of MUs is related to their size (22). We infer small MUs account for the largest proportion in motor neurons pool and the excitability of small MUs exceeds the threshold, let it be the main contributor to Fp. Meanwhile, the excitability of medium and large MU is below the threshold, playing a small part in Fp. However, although less likely, larger MU once activated can produce higher F wave amplitude in view of its size, which is the main reason for higher F/M. Therefore, TA has lower Fp and higher F/M, while the PN is just the opposite.

After stroke, the F/M of TA and PN increased more significant compared to the healthy controls, while Fp did not show any statistical changes. This result is consistent with previous studies (23, 24), and its mechanism had been explained as that the transsynaptic degeneration after stroke may lead to the decrease of the spinal motor neurons, and the size of the remaining MUs increased through collateral budding and re-domination (25). However, this hypothesis cannot explain why Fp did not decrease responding to the declined motor neuron quantity. We proposed there might be other explanations for the different changes of Fp and F/M after stroke. It is believed that the excitability of most spinal motor neurons increased after stroke (11). Because of the high proportion of small MUs in the motor neuron pool and their “pre-saturated” state as high excitability, the increase of excitability after stroke mainly is attributed by larger MUs, which merge with the already excited small MUs, leading to an increased F/M but unchanged Fp.

**Figure 5:**
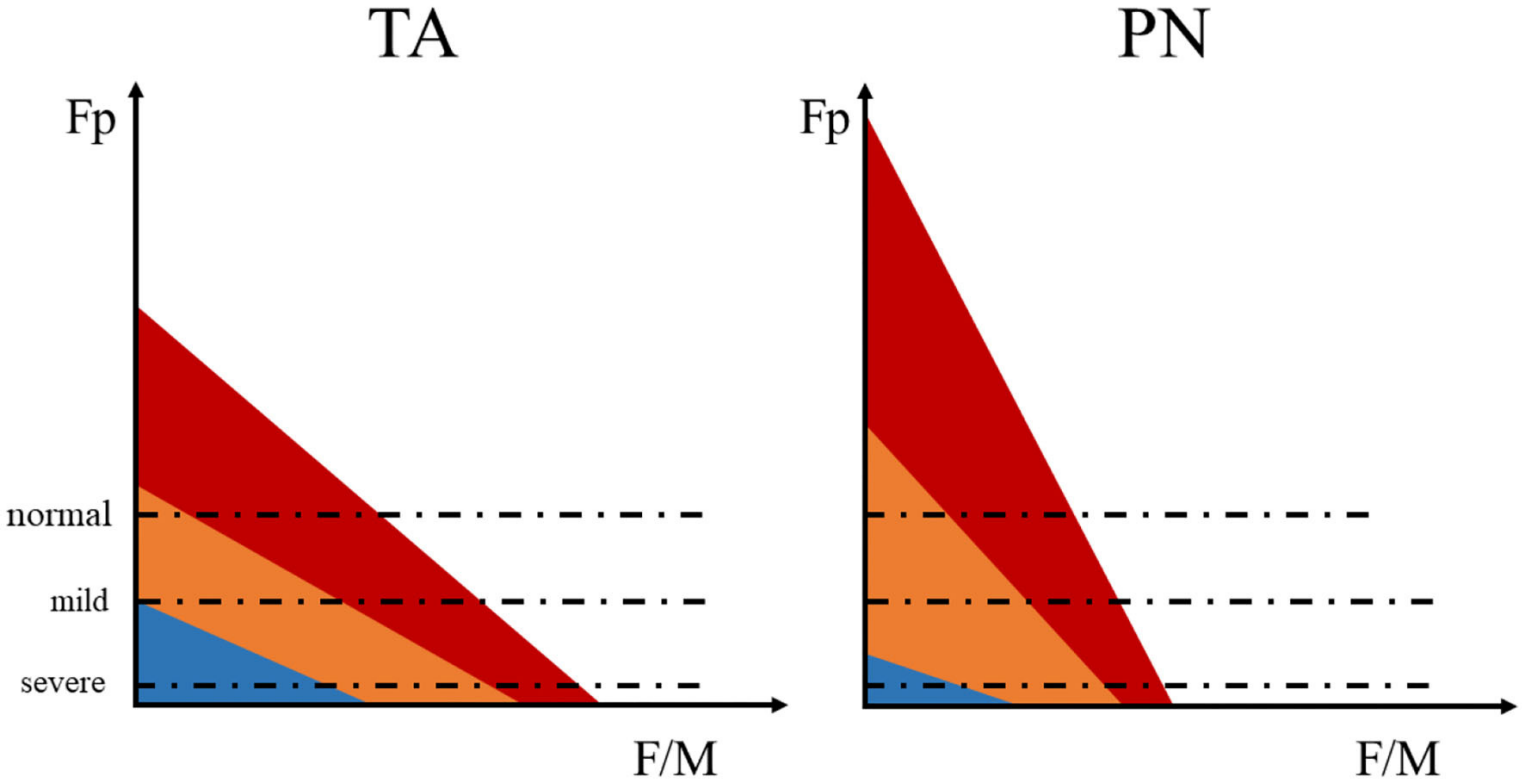
The schematic diagram of the F wave parameters influenced by changed MU excitability. The motor unit (MU) are roughly divided into three types: large MU (blue), medium MU (orange), and small MU (red). The area of each color represents the number of neurons, red > orange > blue. The dashed line represents the relative excitable threshold of the detectable F wave. Excitability exceeding the threshold indicates that the electrical stimulation is more likely to induce a detectable F wave, while excitability below the threshold infer lower probability of detectable F wave under stimulation.

In the purpose of investigating the relation between spinal motor excitability and motor dysfunction after stroke, stratified analysis was performed based on foot inversion and the spasticity of ankle joint. Interestingly, we found the peculiar change of the natural differences between TA and PN's Fp and F/M after stroke. That is, the natural difference of the Fp between TA and PN remained unchanged and F/M's difference disappeared in mild stroke (no/mild spasticity or without foot inversion); while in severe stroke (medium/severe spasticity or with foot inversion), the natural difference between the two muscles remained unchanged in F/M but disappeared in Fp. The reason for this phenomenon might be that in mild stroke, the increased excitability of motor neurons makes the threshold relatively decreased, increasing the probability of F wave generated by medium MU, which had a higher proportion in PN. The newly generated F wave fused with the original F waves predominantly produced by small MUs, leading to larger F waves but unchanged Fp. Thus, the natural difference of F/M between the two muscles disappeared, while the natural Fp difference remained. In severe stroke patients, the excitability of all MUs increased and exceeded the threshold, so the difference in Fp between the two muscles disappeared; and the proportion of large MUs in TA was higher, which could produce F wave with higher amplitude, so the natural differences of F/M between the two muscles reappeared (Figure 5).

Furthermore, to clarify that the change of excitability's difference between the two muscles is the reason for the appearance of foot inversion, the comparison of the change rate of F/M and Fp between two muscles was conducted. The change rate of F/M in TA was higher than PN in patients with foot inversion while no difference showed in the absence of foot inversion. This result indicated that the increased amount of excited large MUs broke the original balance of the two muscles may lead to foot inversion. In addition, when the TA and PN are both paralyzed, the ankle may be manifested as foot inversion under the influence of the gravity and the biomechanics characteristics of the joint. Hereby, we specifically analyzed the patients with ankle dorsiflexion after stroke. In the patients who showed ankle dorsiflexion without foot inversion, there was no significant difference between the TA and PN's F/M, and the Fp of PN was significantly higher. On the contrary, when accompanied by foot inversion, the F/M of TA was significantly higher and Fp showed no difference between the two muscles. According to the result, we can conclude that the change of the excitability difference between the two muscles is still the reason for foot inversion even the biomechanics characteristic of the ankle was considered.

In conclusion, there might be a balance between TA and PN presented as the natural difference in spinal motor excitability. Based on the different proportions of MUs in the two muscles, their excitability may change differently according to the severity of the stroke. So the original balance of excitability would be broken and PN could not fulfill its antagonistic role when TA contracted, leading to foot inversion.

Certainly, there are several limitations in this research. Firstly, using a single F wave test to assess spinal motor neuron excitability is not fully irrefutable. Some researches assumed that F wave cannot precisely reflect the excitability changes of spinal motor neurons considering that higher excitability might lead to the decrease of Fp or F/M (26, 27). Besides, we noticed that pricking was induced in healthy controls during the experiment, but not obviously induced in patients. Previous studies have found that sensory input generated by the electrical stimulus may have some effects on neuron excitability (28, 29). Accordingly, impaired sensory input of the patients will result in a relative decreasing in motor neuron excitability, which may end up with biases of the results. Therefore, studies should take the above limitations into consideration when further test this hypothesis in the future.

## Data Availability Statement

The raw data supporting the conclusions of this article will be made available by the authors, without undue reservation.

## Ethics Statement

The studies involving human participants were reviewed and approved by Huashan hospital Ethics Committee. The patients/participants provided their written informed consent to participate in this study.

## Author Contributions

GL and YW conceived and designed the projects. GL, C-hC, and YCa conducted the experiments. GL and C-hC analyzed the raw data. GL, C-hC, and YCa edited the English. GL, C-hC, X-yS, ST, YCh, X-wT, and R-rL participated in drawing and literature review. All authors contributed clarifications and guidance on the manuscript, involved in editing the manuscript, read, and approved the final manuscript.

## Conflict of Interest

The authors declare that the research was conducted in the absence of any commercial or financial relationships that could be construed as a potential conflict of interest.
